# *In Silico* and *In Vitro* Antimalarial Screening and Validation Targeting *Plasmodium falciparum* Plasmepsin V

**DOI:** 10.3390/molecules27092670

**Published:** 2022-04-21

**Authors:** Xin Ji, Zhensheng Wang, Qianqian Chen, Jingzhong Li, Heng Wang, Zenglei Wang, Lan Yang

**Affiliations:** 1 Institute of Chinese Materia Medica, China Academy of Chinese Medical Sciences, Beijing 100700, China; xinjkaoyan@sina.com; 2 NHC Key Laboratory of Systems Biology of Pathogens, Institute of Pathogen Biology, Chinese Academy of Medical Sciences and Peking Union Medical College, Beijing 100006, China; chenqq2021@outlook.com; 3 Department of Microbiology and Parasitology, Institute of Basic Medical Sciences Chinese Academy of Medical Sciences, School of Basic Medicine Peking Union Medical College, Beijing 100005, China; wangzs@pumc.edu.cn (Z.W.); wangh@ibms.cams.cn (H.W.); 4 NHC Key Laboratory of Echinococcosis Prevention and Control, 21# Linkuo North Road, Chengguan District, Lhasa 850000, China; 13908996200@139.com

**Keywords:** *Plasmodium falciparum*, plasmepsin V, virtual screening, antimalarials, molecular docking

## Abstract

Malaria chemotherapy is greatly threatened by the recent emergence and spread of resistance in the *Plasmodium falciparum* parasite against artemisinins and their partner drugs. Therefore, it is an urgent priority to develop new antimalarials. Plasmepsin V (PMV) is regarded as a superior drug target for its essential role in protein export. In this study, we performed virtual screening based on homology modeling of PMV structure, molecular docking and pharmacophore model analysis against a library with 1,535,478 compounds, which yielded 233 hits. Their antimalarial activities were assessed amongst four non-peptidomimetic compounds that demonstrated the promising inhibition of parasite growth, with mean IC_50_ values of 6.67 μM, 5.10 μM, 12.55 μM and 8.31 μM. No significant affection to the viability of L929 cells was detected in these candidates. These four compounds displayed strong binding activities with the *Pf*PMV model through H-bond, hydrophobic, halogen bond or π-π interactions in molecular docking, with binding scores under −9.0 kcal/mol. The experimental validation of molecule-protein interaction identified the binding of four compounds with multiple plasmepsins; however, only compound **47** showed interaction with plasmepsin V, which exhibited the potential to be developed as an active *Pf*PMV inhibitor.

## 1. Introduction

Malaria is a pernicious parasitic disease that has tortured human beings since ancient times, and it remains a major health problem for people in tropical and subtropical regions. According to the latest *World Malaria Report*, there were an estimated 241 million malaria cases and 627,000 malaria deaths worldwide in 2020 [1]. The Plasmodium parasites, including *Plasmodium falciparum*, *P. vivax*, *P. ovale*, *P. malaroae* and *P. knowlesi*, are culprits of human malaria. *P. falciparum* is the most virulent species that accounts for the majority of mortality, especially in children under five years old and in pregnant women. *P. vivax* is the most geographically widespread parasite that can cause relapsing episodes, which can substantially increase its morbidity [2]. Clinical malaria treatment relies heavily on effective chemotherapy; nevertheless, the emergence and spread of resistance to antimalarial drugs jeopardize malaria control and elimination. Because of the rapid development of resistance, particularly in *P. falciparum,* to multiple drugs, including chloroquine, sulfadoxine-pyrimethamine, mefloquine and quinine, artemisinin-based combination therapies (ACTs) have been recommended as the frontline treatment of falciparum malaria since 2005 to avoid the development of resistance to artemisinin and its derivatives (ARTs). As the cornerstone of ACTs, ARTs are active against a large range of intraerythrocytic parasite developmental stages and play an integral role in reducing the global malaria burden [3]. To our dismay, clinical resistance to ARTs has emerged in *P. falciparum* and is gradually spreading in Southeast Asia [4,5,6]. Adding to the woe, the waning efficacy of dihydroartemisinin/piperaquine in Cambodia implies that parasites show resistance to the combination regimens [7,8]. As a result, malaria chemotherapy is confronting serious challenges because no replacement drug is available. Therefore, it is an urgent priority to replenish antimalarial stock to prevent the resurgence of malaria. Equally necessary is the concurrent need to mine the parasite genome and aim at those “druggable” targets to identify antimalarial candidates.

Of the potential targets for exploitation in drug discovery, plasmodium proteases gain more attention due to their roles in various parasite developmental activities and their well-characterized mechanisms and structures. Amongst the plasmepsins are aspartic proteases involved in hemoglobin degradation and other crucial processes [9]. The *P. falciparum* genome encodes for 10 different isoforms of this family (plasmepsin I to X) [10]. Of these, plasmepsin V (*Pf*PMV) is considered a superior drug target as it functions in processing malarial proteins to transport from the intra-erythrocytic parasite to the red blood cell cytosol [11]. The *P. falciparum* parasite exports over 450 proteins into the host erythrocyte to remodel the cytoskeleton and plasma membrane for cytoadherence and nutrient permeation pathways. The export is dependent on the cleavage of a specific sequence motif called PEXEL (Plasmodium EXport ELement) [12,13]. *Pf*PMV acts as a gatekeeper that cleaves the PEXEL at the C-terminus side of the conserved Leu and licenses the proteins destined for export [14,15,16].

Given that *Pf*PMV plays an indispensable role in parasite development, an increasing number of studies focus on the identification of its inhibitors. The well-known class are the homology compounds that mimic the natural PEXEL substrate, represented by WEHI-916, WEHI-842 and WEHI-601 that inhibit *Pf*PMV at nanomolar [11,17,18], as well as an analogue compound at picomolar [19]. However, these inhibitors act poorly against parasite growth, with the lowest IC_50_ value around 15μM. This is possibly due to the poor stability of their peptidic components and trans-membrane permeability [19]. Consequently, targeting *Pf*PMV to screen new chemical scaffolds with better antimalarial performance is still needed.

In the present study, we used virtual screening techniques based on a combination of homology modeling of the protein crystal structure and pharmacophore analysis to screen small molecule inhibitors throughout the commercial compound library. Through in vitro assessment of the hit compounds against *P. falciparum* parasite development and Drug Affinity Responsive Target Stability (DARTS) validation, we report new antimalarial candidates to potentially target *Pf*PMV and other plasmepsin proteins.

## 2. Results

### 2.1. Homology Modeling of PfPMV and Virtual Screening

**Figure 1:**
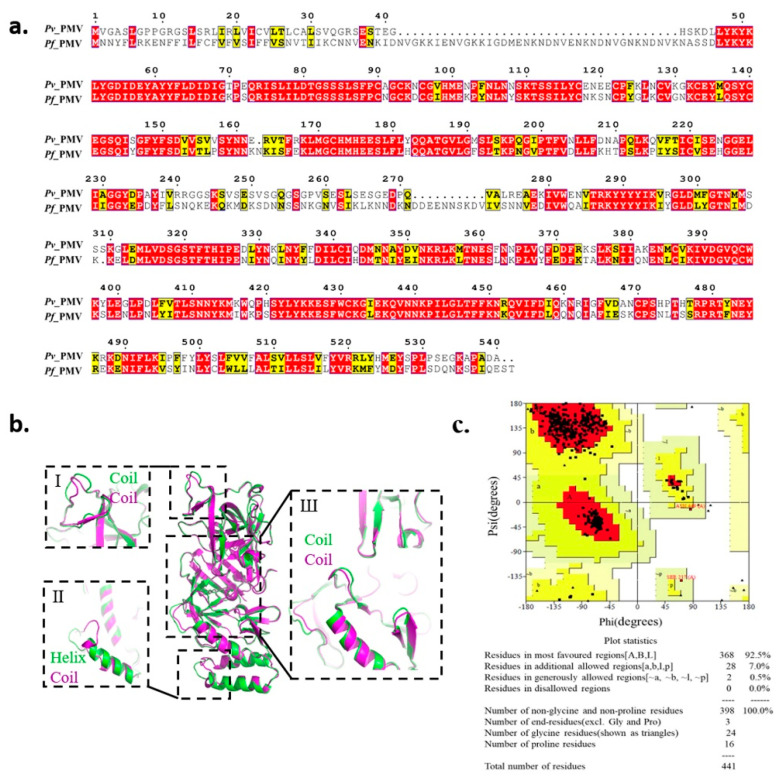
Homology modeling of *Pf*PMV 3D structure. (**a**) Sequence comparison of plasmepsin V in *Plasmodium vivax* and *Plasmodium falciparum*. (**b**) Superposition of homology 3D structure of *Pf*PMV (M09, in purple) and the crystal structure of *Pv*PMV (PDB: 4ZL4, in green). (**c**) The Ramachandran plot of model M09.

Since a crystal structure of *Pf*PMV is not yet available on the Protein Data Bank (PDB), we modeled its 3D structure based on the recently obtained X-ray crystal structure of *Pv*PMV bound to WEHI-842 (PDB: 4ZL4) [18]. PMV showed to be conserved in different strains of *P. falciparum* with various origins (Dd2 from Southeast Asia, 7G8 from Brazil, South America, and HB3 from Honduras, South America) as compared with the standard laboratory isolate 3D7, with the sequence identities ranging from 99.32% to 100% (Figure S1). Therefore, we used the sequence from 3D7 for further analysis, which showed 67.9% of the sequence identity to the *Pv*PMV sequence (Figure 1a). A total of 20 models were constructed (Table S1), and the model (M09) with the best scores was selected based on the assessment of the Pair Distribution Function (PDF) Total Energy, PDF Physical Energy and Discrete Optimized Protein Energy (DOPE) Score. As illustrated by the superposition of *Pv*PMV and *Pf*PMV model M09 in Figure 1b, the two proteins were generally superimposed well, except for three folding differences at positions I, II and III. As shown at position I, the coil was irregular in *Pf*PMV compared with that in *Pv*PMV. A major variance was noticed at position II, where *Pv*PMV folded in a helix while *Pf*PMV was in a coil. Slight inconsistency was also observed at position III, which is located around the active pocket. The Ramachandran plot analysis showed that 92.5% of the amino acid residues in this model were in the most favored regions, 7.5% were in additional or generously allowed regions and no residue was in disallowed regions (Figure 1c), indicating a suitable environment for each amino acid residue in the structure. This homology model was employed for virtual screening to test the commercial chemical library ChemDiv2019 containing 1,535,478 small molecule compounds. A total of 1507 compounds were retrieved by molecular docking.

**Figure 2:**
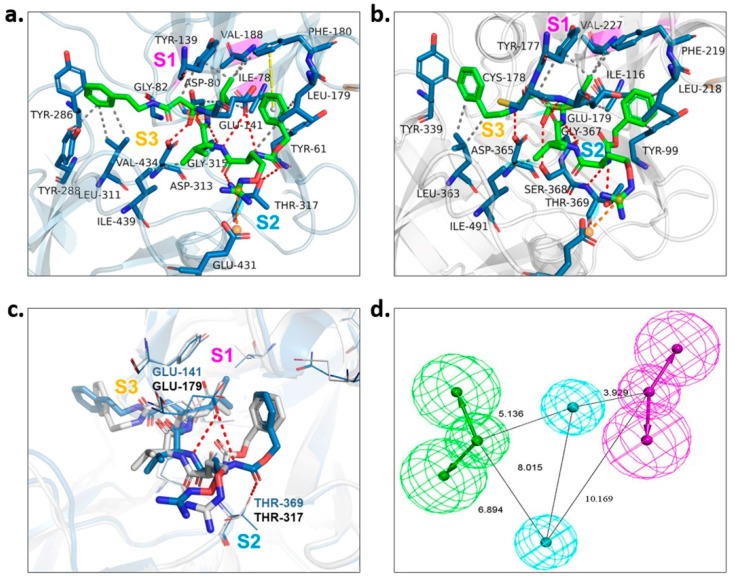
Pharmacophore model construction. (**a**) Protein–ligand interactions of WEHI-842 with *Pv*PMV. S1, S2 and S3 represent three subpockets. WEHI-842 is colored green. Dashed line in red indicates H-bond interactions, grey represents hydrophobic interaction, yellow illustrates pi–pi interaction, and orange implies salt bridge interaction. (**b**) Protein–ligand interactions of WEHI-842 with *Pf*PMV (M09). (**c**) Superposition of *Pf*PMV (in grey) and *Pv*PMV (in light blue) interacted with WEHI-842. (**d**) The pharmacophore model of *Pf*PMV.

To gain the best hits, screening based on pharmacophore model analysis was performed simultaneously. According to the investigation of the substrate-binding sites of *Pv*PMV in complex with WEHI-842 [17] (Figure 2a) and the structure-activity relationship of the PEXEL peptidomimetics [9,11], the -OH group which forms an H-bond interaction with Asp 80 and Asp 313 is important to the inhibition activity. The S1 pocket in *Pv*PMV is composed of hydrophobic amino acid residues (Ile78, Val188 and Tyr139) [17], which require the binding of compounds with hydrophobic groups. The S2 binding cavity is lined by mostly hydrophobic amino acid residues (Ile439, Val434, Tyr286, Tyr288, Leu311 and Thr317) as well. Previous findings indicated that fully occupying the S2 subpocket could greatly increase the activity against PMV [9]. In addition, the guanidino group that forms the H-bond interaction with Glu141 and Gln183 in the S3 subpocket might also contribute to the inhibition [11]. The molecular docking of WEHI-842 to the homology 3D model M09 showed the active sites of *Pf*PMV. Correspondingly, the S1 subpocket consisted of hydrophobic amino acid residues Val227, Tyr177 and Ile116; the S2 subpocket included residues ASP365, Thr369 and SER368 that could form an H-bond interaction with the active compound, and the S3 pocket was composed of Glu179 and Gly367 to form H-bond interactions (Figure 2c). However, minor differences were observed in the superposition of WEHI-842 interacting with both *Pv*PMV and *Pf*PMV models, where two amide groups formed H-bond interactions with the residue of Glu141 in the S2 subpocket and Thr369 in the S3 subpocket of *Pv*PMV, respectively. This was not noticed in *Pf*PMV, which might be due to the differences in residue side chains in these two proteins. Consequently, the pharmacophore model was constructed to possess two H-bond acceptors, two H-bond donors and two hydrophobic centers (Figure 2b). Virtual screening utilizing this model output 189 compounds.

Therefore, the combination of the results from both approaches gave rise to a total of 1664 compounds, with an intersection of 32 chemicals. These compounds were further screened to yield the top 15% hits with higher docking scores. Consequently, 249 compounds were retrieved, and 233 were commercially available as final candidates for the in vitro anti-malaria susceptibility assay.

### 2.2. Anti-Malaria Activity and Cytotoxicity of Candidate Compounds

**Figure 3:**
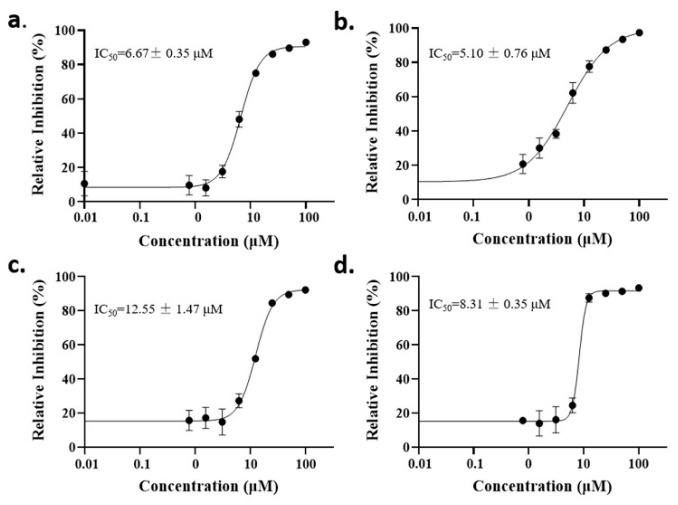
Dose–response curves of candidate compounds. (**a**–**d**) represent the dose–response curve of compounds **17**, **47**, **62** and **147**.

**Figure 4:**
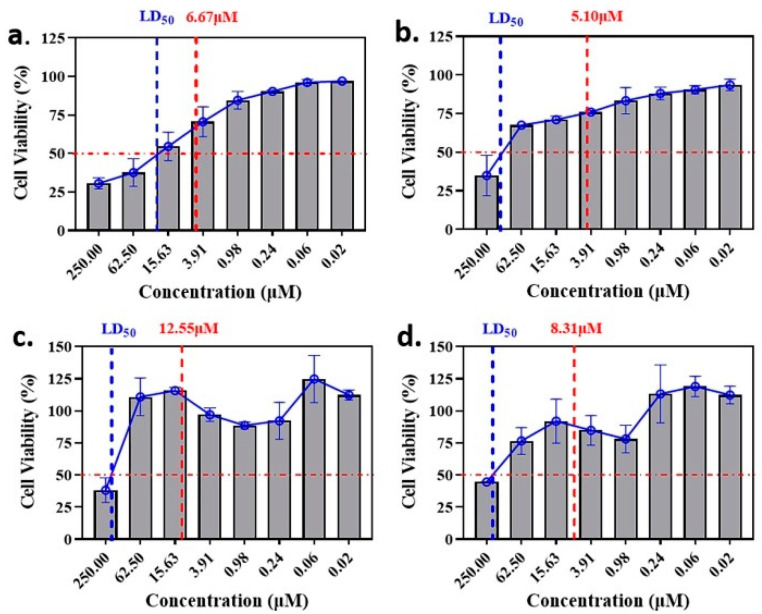
Comparison of LD_50_ and IC_50_ values of the candidate compounds. (**a**–**d**) indicate the comparison for compounds **17**, **47**, **62** and **147**.

The anti-malaria activities of the final hits from virtual screening were tested employing the laboratory strain *P. falciparum* 3D7. Four alkaloid compounds, **17**, **47**, **62** and **147**, showed promising inhibition of parasite growth, with mean IC_50_ values of 6.67 μM, 5.10 μM, 12.55 μM and 8.31 μM (Figure 3, Table 1). These candidates were more active against parasite development than those PEXEL analogues [11,17,18]. The potential cell cytotoxicity was subsequently assessed using the L929 cell line, as recommended by the International Organization for Standardization [20]. The four chemicals demonstrated no significant effect on the cell viability of L929 cells, with the lowest LD_50_ value above 28 μM, much higher than their IC_50_ values against *P. falciparum* 3D7 (Figure 4).

### 2.3. Binding Target Investigation of Candidate Compounds

**Figure 5:**
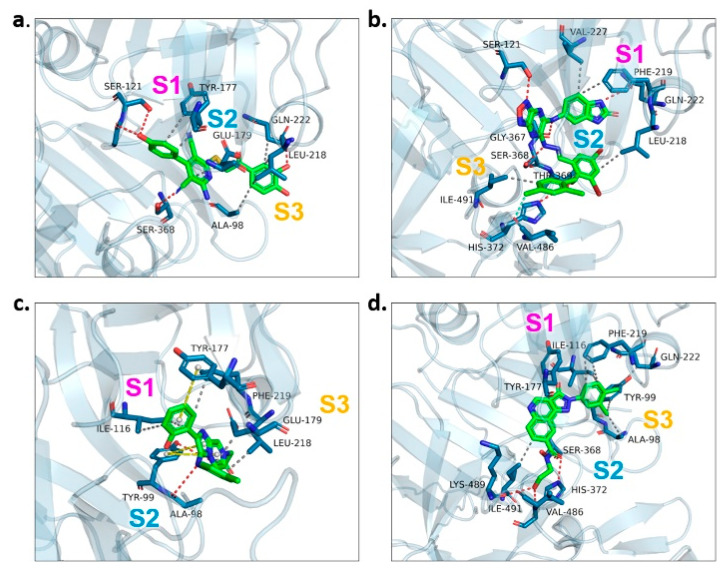
Proteinligand interactions of candidate compounds with *Pf*PMV. (**a**–**d**) illustrate the binding of residues with compounds **17**, **47**, **62** and **147**. Candidate compounds were shown in green color. S1, S2 and S3 represent three subpockets. Dashed line in red indicates H-bonds interactions, grey represents t hydrophobic interaction, yellow illustrates pi–pi interaction and blue implies halogen bond interaction.

We examined the amino acid residue contribution of the *Pf*PMV active sites to four candidate inhibitors by docking these compounds to the homology 3D model. A binding score under −1.5 kcal/mol was considered to have potential binding activity [21]. All of the four compounds showed values under −9.0 kcal/mol (Table 1), indicating they might have strong binding activities with *Pf*PMV. The binding complex of **17** and *Pf*PMV illustrated that two hydroxyl and alkynyl substitutes of this molecule formed H-bond interactions with Ser-121, Gln-222 and Ser-368, and two benzene rings formed hydrophobic interactions with Tyr-177, Glu-179, Ala-98, Leu-218 and Gln-222. Five nitrogen groups in compound **47** formed H-bond interactions with Ser-121, Ser-38, His-372, Gly-367 and Gln-222. Hydrophobic interactions with Ile-491, Leu-218, Phe-219 and Val-227 were basically formed by three benzene rings. In addition, one bromo group formed a halogen bond with Val-486. In Compound **62**, two nitrogen groups formed H-bond interactions with Tyr-99 and Ala-98. Hydrophobic interactions with Ike-116, Leu-218, Glu-179 and Phe-219. A benzene ring was also shown to make the π-π interaction with Tyr-177. The hydroxy and carboxide on the side chain of **147** formed H-bonds with Lys-489, Val-486 and His-372, and the benzene rings formed hydrophobic interactions with Ile-491, Tyr-177, Ile-116, Phe-219, Gln-222, Tyr-99 and Ala-98 (Figure 5, Table 1). All three subpockets (S1, S2 and S3) in *Pf*PMV were occupied by **47**, **62** and **147**, while the S2 region in the binding complex of **17** was not taken.

**Figure 6:**
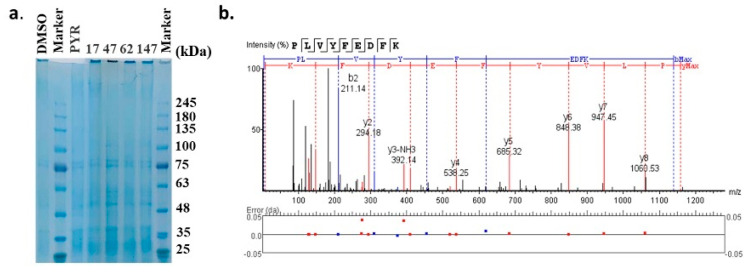
Target validation of candidate compounds by DARTS strategy. (**a**) SDS−PAGE gel of the compound−protein complexes. (**b**) Identification of the plasmepsin V based on the label−free LC−MS/MS analysis.

The molecular targets of these compounds were further experimentally validated via the DARTS strategy, a universally applicable target identification approach that analyzes direct drug binding to targets. The compound–protein complexes were examined by SDS-PAGE gel (Figure 6a) and analyzed through label-free LC-MS. The top 20 proteins that potentially interacted with each compound are displayed in Table 2, indicated by the maximum area of the peptide from each protein. Pyrimethamine was exploited as a reference for its known interaction with the dihydrofolate reductase-thymidylate synthase (DHFR-TS) protein, which showed the maximum area of the peptide as 1.54 × 10^7^. DHFR-TS were also identified as interacting with four candidates, as well as chloroquine resistance transporter (CRT), a member of the drug/metabolite transporter superfamily located on the membrane of food vacuole [22]. Interestingly, all of the four candidates exhibited binding activities with plasmepsin I-IV, the aspartic proteases involved in the hemoglobin metabolism inside the parasite food vacuole [23]. However, only **47** was detected bound with plasmepsin V, implied by the detection of peptide KPLVYFEDFKT with a maximum area of 6.21 × 10^6^ (Figure 6b, Table 2). Since the DARTS method has potential limitations, further validation based on protein manipulation could possibly provide more evidence.

## 3. Discussion

Although malaria control has made great strides in recent decades, the recent emergence of resistance to ARTs and their partner drugs has become a major concern. The Discovery of new antimalarial drugs is therefore desperately needed. PMV resides in the endoplasmic reticulum and is a key aspartate protease required to export proteins to the host cell by cleaving the conserved motif PEXEL, thus making this enzyme an important drug target. Previous inhibition strategies focused on the synthesis of peptidic compounds that physically resemble the PEXEL but cannot be cleaved by PMV [9]. These peptides bind to and block the active site of PMV, hence interrupting the cleavage of PEXEL and suspending the export of parasite proteins, such as the key virulence protein *Pf*EMP1. However, this type of inhibitor showed weak inhibition to parasite growth, possibly due to its low membrane permeation ability or ease to be degraded by other enzymes. Therefore, this study concentrated on the detection of non-peptidomimetic drug-candidate inhibitors with new scaffolds.

In silico approaches support drug discovery in a time and cost-effective manner. Despite its critical importance and potential as a drug target, *Pf*PMV still lacks a 3D structure. *Pv*PMV crystal structure is reported as a drug complex with WEHI-842 (PDB 4ZL4) [17] or with WEHI-601 (PDB 6C4G) [18]. However, the homology 3D structure of *Pf*PMV could be modeled on the basis of *Pv*PMV since the two proteins have 67.9% of the sequence identity [24,25]. In addition to commonly used molecular docking, virtual screening employing structure-based pharmacophore derived from interactions of the known active compound with a 3D model of target protein remains an attractive way; we thus applied both methods to improve the hit rate.

Interestingly, four candidate compounds identified in the present study inhibited *P. falciparum* parasite development at low micromolar concentrations, performing much better than previously reported PEXEL peptidomimetics [18]. Evidence from molecular docking illustrated that the S2 subpocket of the *Pf*PMV complex was not taken by **17**; however, further molecular modification could focus on the chain of this compound to form hydrophobic interactions with residues in S2 and improve its antimalarial activity. In contrast, the active sites of *Pf*PMV were fully occupied by compounds **47**, **62** and **147**, indicating strong interactions of them with the protein. The binding affinity of these candidates was validated by DARTS. This method takes advantage of a reduction in the protease susceptibility of the target protein upon drug binding and requires no modification of the drug; thus, it is universally applicable [26]. The LC-MS recognized a series of proteins, with the majority in the pathways of energy transport. It is possible that some of these proteins work in complex and thus are identified collectively. For example, the food vacuole plasmepsin I to IV were detected bound with all four compounds, as well as for CRT, a drug/metabolite transporter located on the membrane of food vacuole, which confers chloroquine resistance by exporting the drug. However, only **47** was identified to interact with *Pf*PMV in DARTS. Likewise, this compound shows the most effective activity against *P. falciparum*, which has the potential to be further developed as a promising *Pf*PMV inhibitor. Since a fundamental limitation for DARTS is that the susceptibility of a protein to proteolysis is determined by its conformational energy landscape, it is possible that the stability of the complex with **17**, **62** and **147** might affect the detection. Therefore, further investigation through gene manipulation to verify the binding is needed.

## 4. Materials and Methods

### 4.1. Homology Modeling of PfPMV 3D Structure

The *Pf*PMV (PF3D7_1323500) amino acid sequence from the standard laboratory strain 3D7 was downloaded from PlasmoDB [27], along with sequences from isolates Dd2, 7G8 and Hb3. Their sequence identity was evaluated through the ESPript server [28]. The recently obtained X-ray crystal structure of *Pv*PMV bound to WEHI-842 (PDB: 4ZL4) [18] was used as a template to model the *Pf*PMV 3D structure. The models were constructed in Discovery Studio 2016. The stereochemistry of the final model was further evaluated via the Verify Protein (Profiles-3D) module in Discovery Studio 2016 [29], and the Ramachandran plot was analyzed using the Procheck server [30].

### 4.2. Virtual Screening through Molecular Docking

The software Schrödinger 2015 (Schrödinger, LLC, New York, NY, USA, 2015) was used for the virtual screening of the ChemDiv2019 compound library. The *LigPrep* module (Schrödinger Release 2015-1: LigPrep, Schrödinger, LLC, New York, NY, USA, 2015) was employed to process the library, with the force field set to OPLS3 [31] and other parameters as default. Three modes in the module virtual Screening Workflow were used for further screening through flexible docking. The library was initially screened at the mode of Glide HTVS [32], and the top 10% compounds based on the docking scores were next processed at Glide XP mode to select the top 10% compounds in this group, and these compounds were finally selected via Glide XP mode to output the top 10% compounds.

### 4.3. Virtual Screening by Pharmacophore Model Analysis

The protein–ligand interactions of WEHI-842 with *Pv*PMV were converted to a pharmacophore model by Discovery Studio 2016. The ChemDiv2019 compound library was processed by the Build 3D Database module. Screening was performed using the Search 3D Database module, followed by pharmacophore docking with three modes. The top 50% compounds selected by mode Glide HTVS were applied to mode Glide SP, and the top 50% compounds of this group were input for Glide XP mode to obtain the top 10% compounds as final output.

### 4.4. In Vitro P. falciparum Parasite Culture and Susceptibility Assay

The standard laboratory *P. falciparum* strain 3D7 was used for the in vitro antimalarial sensitivity assay. Parasites were cultured as previously described [33]. Briefly, the isolate 3D7 was maintained within fresh human red blood cells (RBCs) at 5% hematocrit in RPMI-1640 medium supplemented with 0.5% (*w*/*v*) albumax II (Invitrogen, Carlsbad, CA, USA) under an atmosphere of 90% N_2_, 5% O_2_ and 5% CO_2_ at 37 °C. Parasitemia was monitored by Giemsa staining of blood smears. A standard SYBR Green I-based fluorescence assay was used to assess parasite susceptibilities to candidate compounds as reported in [34]. The 233 candidate inhibitors were purchased from Topscience Inc. (Shanghai, China). The stock solution of compounds was dissolved in dimethyl sulfoxide (DMSO) and further diluted by complete medium to a final concentration of 200 µM. Cultures were synchronized by 5% D-sorbitol treatment twice, and ring-stage parasites were assayed in 96-well microtiter plates at 1% hematocrit and 0.5% parasitemia. Three technical repeats and three biological replications were performed for each sample concentration. DMSO at a concentration of 0.1% (*v*/*v*) was used as a negative control. IC_50_ values were measured using a non-linear regression model in GraphPad Prism 7 (GraphPad Software, Inc., La Jolla, CA, USA).

### 4.5. Culture of L929 Cell Line and Cell Viability Assessment

The murine fibroblast (L929) cell line was purchased from the National Biomedical Cell-line Resource Center (Beijing, China) and maintained in Dulbecco’s modified Eagle medium (DMEM, Thermo Fisher, Waltham, MA, USA) supplemented with 10% FBS (Gibco, Carlsbad, CA, USA) in a humidified atmosphere at 37 °C with 5% CO_2_. The cell viability of each compound was assessed via a CCK-8 kit (Beijing Solarbio Science & Technology Co., Ltd., Beijing, China) following the manufacturer’s instructions. Briefly, L929 cells were counted and dispersed into 96-well plates at 100 µL per well. After 48 h incubation, 10 µL of each compound at 2.75 mM was added and incubated for 24 h. Afterward, 10 µL of CCK-8 solution was added to each well and incubated for 4 h. A microplate reader (Infinite M200, Tecan Group Ltd., Männedorf, ZH, Switzerland) was employed to measure the absorbance of the cells at 450 nm.

### 4.6. Molecular Docking of Candidate Compounds to PfPMV Active Sites

The 3D structures of the candidate compounds were constructed using Chem 3D v20 and energy-minimized under the MMFF94 force field. The AutoDock Vina 1.1.2 (Scripps, San Diego, CA, USA) software [35] was utilized for docking, with the maximum number of evals of genetic algorithm parameters set to 32 and the remaining parameters at default. The docking complexes were visualized by PyMol 2.5 (Schrödinger, New York, NY, USA) [36].

### 4.7. DARTS Experiment for Target Investigation

The *P. falciparum* protein was extracted according to the literature [37]. The protein concentration was determined using a BCA Protein Concentration Assay Kit (Beijing Solarbio Science & Technology Co., Ltd., Beijing, China), and the protein solution was diluted to 2 μg/μL for later use. The DARTS experiment was performed referring to the reported protocols [26,38]. Briefly, 5 μL of each candidate compound was suspended in 45 μL of protein solution to reach a final concentration of 1 mM. This was mixed and incubated for 30 min at room temperature, with 10% (*v*/*v*) DMSO processing in the same way as a negative control. Subsequently, 2 μL of pronase from *Streptomyces griseus* (pronase/protein 1:800, Merck & Co., Inc., Kenilworth, NJ, USA) was added and incubated for 15 min, followed by adding 2 μL of the protease inhibitor mixture (Beyotime Biotechnology, Shanghai, China) and incubating on ice for 10 min to terminate the reaction. The reaction solution was checked using SDS-PAGE gel electrophoresis and then applied to protein characterization by label-free LC-MS (Themofisher, Waltham, MA, USA). The tandem mass spectra were analyzed by PEAKS Studio 10.6 (Bioinformatics Solutions Inc., Waterloo, ON, Canada) and annotated based on the database of uniprot-*Plasmodium falciparum* (3D7) using the module PEAKS DB (version 202201, 5380 entries).

## 5. Conclusions

This study performed virtual screening for small molecules by both molecular docking and pharmacophore analysis approaches. Four potent inhibitors of *Pf*PMV with higher activities against *P. falciparum* parasites were identified. The effective antimalarial activity and low cytotoxicity of the four candidates largely suggested these chemicals may be considered for further structural optimization studies and subsequent experimental validations. Our findings showed that the inhibition of *Pf*PMV activity by non-peptidomimetic inhibitors could potentially be further developed into a more potent PMV inhibitor.

## Figures and Tables

**Table 1:** List of molecules on the basis of docking score (kcal/mol) and interacting residues of *Pf*PMV with four candidate compounds.

Candidate Compound	Molecule	H-Bonds	Hydrophobic Interactions	Halogen Bond	π-π Interaction	Docking Score
**17**	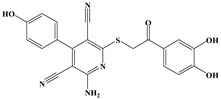	Ser-121, Gln-222, Ser-368	Tyr-177, Glu-179, Ala-98, Leu-218, Gln-222	-	-	−9.3
**47**	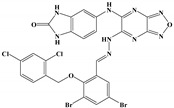	Ser-121, Ser-38, His-372, Gly-367, Gln-222	Ile-491, Leu-218, Phe-219, Val-227	Val-486	-	−10.9
**62**	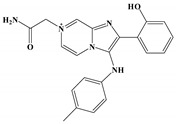	Tyr-99, Ala-98	Ike-116, Leu-218, Glu-179, Phe-219	-	Tyr-177	−9.3
**147**	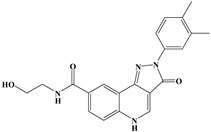	Lys-489, Val-486, His-372	Ile-491, Tyr-177, Ile-116, Phe-219, Gln-222, Tyr-99, Ala-98	-	-	−10.4

**Table 2:** The top 20 proteins detected by DARTS and the maximum area of the peptide from the corresponding protein.

Identified Protein	Peptide Maximum Area			
	17	47	62	147
Ornithine aminotransferase	6.08 × 10^8^	7.88 × 10^8^	7.36 × 10^8^	6.20 × 10^8^
Phosphoglycerate kinase	4.89 × 10^8^	4.53 × 10^8^	3.94 × 10^8^	4.16 × 10^8^
Plasmepsin Ⅲ	2.73 × 10^8^	1.72 × 10^8^	2.39 × 10^8^	2.29 × 10^8^
Plasmepsin Ⅳ	2.13 × 10^8^	2.08 × 10^8^	1.96 × 10^8^	2.02 × 10^8^
Plasmepsin Ⅱ	1.76 × 10^8^	2.08 × 10^8^	1.96 × 10^8^	2.02 × 10^8^
Adenosine deaminase	1.67 × 10^8^	1.89 × 10^8^	1.31 × 10^8^	1.43 × 10^8^
Adenosylhomocysteinase	1.14 × 10^8^	1.23 × 10^8^	1.03 × 10^8^	7.73 × 10^7^
Pyridoxine biosynthesis protein PDX1	6.94 × 10^7^	9.19 × 10^7^	9.71 × 10^7^	3.15 × 10^7^
Plasmepsin Ⅰ	8.21 × 10^7^	9.14 × 10^7^	8.67 × 10^7^	4.36 × 10^7^
Serine repeat antigen 5	3.22 × 10^7^	4.73 × 10^7^	3.49 × 10^7^	7.73 × 10^7^
Proliferating cell nuclear antigen	3.74 × 10^7^	7.45 × 10^7^	3.75 × 10^7^	4.39 × 10^7^
Surface protein P113	4.09 × 10^7^	3.57 × 10^7^	3.55 × 10^7^	4.30 × 10^7^
6-cysteine protein PF92	3.21 × 10^7^	2.27 × 10^7^	2.88 × 10^7^	2.88 × 10^7^
Serine repeat antigen 6	2.33 × 10^7^	1.94 × 10^7^	1.77 × 10^7^	2.28 × 10^7^
Bifunctional dihydrofolate reductase-thymidylate synthase	1.58 × 10^7^	1.85 × 10^7^	1.56 × 10^7^	1.62 × 10^7^
DNA repair protein RAD50	1.10 × 10^7^	1.55 × 10^7^	ND	1.74 × 10^7^
Chloroquine resistance transporter	1.08 × 10^7^	1.38 × 10^7^	6.74 × 10^6^	1.13 × 10^7^
Prefoldin subunit 6	1.37 × 10^7^	ND	1.16 × 10^7^	1.20 × 10^7^
Myosin A	7.16 × 10^6^	7.70 × 10^6^	7.10 × 10^6^	6.57 × 10^6^
Plasmepsin V	ND	6.21 × 10^6^	ND	ND

Note: ND means not detected.

## Data Availability

Not applicable.

## Supplementary Materials

The following supporting information can be downloaded at: https://www.mdpi.com/article/10.3390/molecules27092670/s1. Table S1: Predicted homology 3D models of *Pf*PMV and their evaluation scores. Figure S1: Sequence identifies of PMV in different strains of *P. falciparum* with various origins.

## Sample Availability

Samples of the compounds are not available from the authors.

## Abbreviations

ARTs	Artemisinin and its derivatives
ACTs	Artemisinin-based combination therapies
PMV	Plasmepsin V
*Pf*PMV	*Plasmodium falciparum* Plasmepsin V
*Pv*PMV	*Plasmodium vivax* Plasmepsin V
PEXEL	Plasmodium EXport ELement
IC_50_	Half maximal inhibitory concentration
LD_50_	Lethal dose of 50%
DARTS	Drug Affinity Responsive Target Stability
DMSO	Dimethyl sulfoxide
