# Supplementary material for: In Silico and In Vitro Antimalarial Screening and Validation Targeting Plasmodium falciparum Plasmepsin V
Source: Molecules. 2022 Apr 21;27(9):2670. doi: 10.3390/molecules27092670 (PMC9102085; doi:10.3390/molecules27092670)
Supplement: Supplementary file 1 [file molecules-27-02670-s001.zip › molecules-1680764-supplementary.pdf]

Table S1. Predicted homology 3D models of *Pf*PMV and their evaluation scores.

Model Scores			
**Name**	**PDF Total Energy**	**PDF Physical Energy**	**DOPE Score**
M0009	2780.0994	1365.3197299	−49870.484375
M0010	2816.1169	1388.4467379	−49577.097656
M0007	2893.9834	1477.979074	−48868.937500
M0019	2941.2268	1429.8381153	−49394.183594
M0006	2977.6260	1454.2111707	−49437.207031
M0001	2985.6101	1397.591110397	−49241.277344
M0003	3006.6924	1507.5281204	−49195.449219
M0012	3046.7537	1480.114227336	−49480.691406
M0002	3104.5000	1512.04063713	−49481.757813
M0018	3116.6721	1531.3116855	−49085.625000
M0011	3131.6362	1517.60372829999	−48924.609375
M0016	3165.6443	1718.1146458	−49442.320313
M0015	3179.0183	1522.113210415	−49679.648438
M0013	3212.2351	1749.70724256	−49367.039063
M0014	3238.7507	1484.36043	−50293.988281
M0020	3297.6702	1562.7906949	−48747.710938
M0008	3808.0076	1896.4823133177	−49550.777344
M0004	5311.2568	1854.401087916	−48715.539063
M0017	5496.1753	1940.85879527	−49142.046875
M0005	5657.1245	2062.600143	−49755.136719

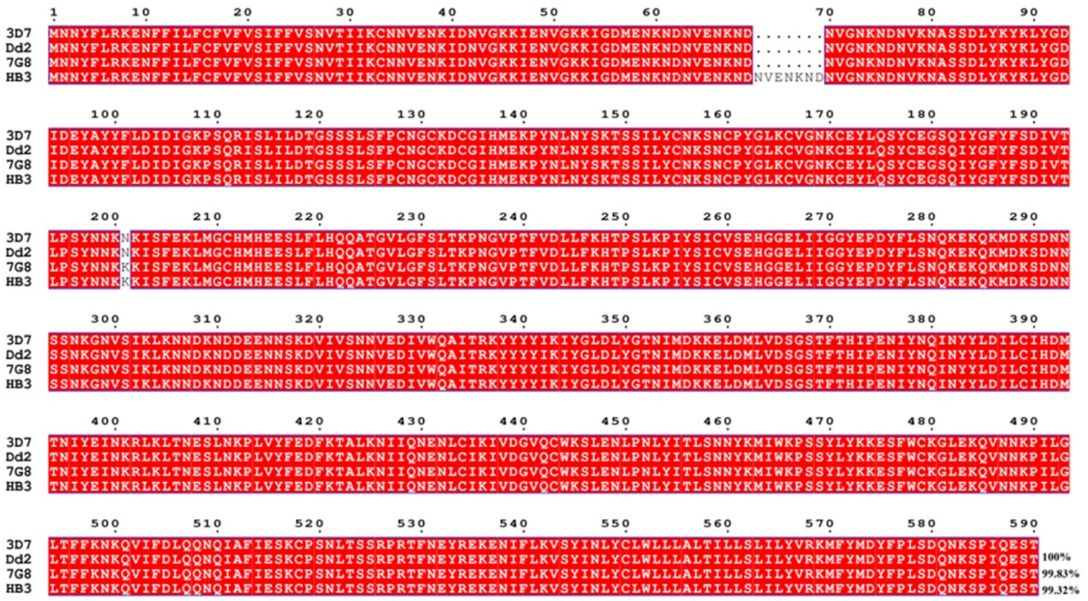

Figure S1. Sequence identifies of PMV in different strains of *P. falciparum* with various origins.
